# Rationale for the Use of Homologous Recombination Proficient Molecular Profile as a Biomarker for Therapeutic Targeting in Ovarian Cancer

**DOI:** 10.3389/or.2023.11471

**Published:** 2023-09-20

**Authors:** John Nemunaitis, Laura Stanbery, Adam Walter, Rodney Rocconi, Philip Stephens

**Affiliations:** ^1^ Gradalis, Inc, Dallas, TX, United States; ^2^ ProMedica, Toledo, OH, United States; ^3^ Division of Gynecologic Oncology, Department of Obstetrics and Gynecology, University of Alabama at Birmingham, Mobile, AL, United States; ^4^ Naveris, Waltham, MA, United States

**Keywords:** homologous recombination, neoantigen, immune response, ovarian cancer, immunotherapy

## Introduction

Therapeutic options for advanced-stage ovarian cancer patients are limited in those subjects with homologous recombination proficient molecular profiles. A recent review of the existing literature demonstrates evidence of enhanced relapse-free survival and overall survival associated with treatment with Vigil in the Phase 2b trial in the HRP population. Homologous recombination (HR) is a genetic rearrangement in which molecular information is exchanged between two similar molecules of double-stranded or single-stranded nucleic acids [1]. The purpose of HR is to maintain genome stability by performing high-fidelity repair of complex DNA damage such as DNA double-strand breaks and interstrand crosslinks [2–4].

Homologous recombination is responsible for double-stranded DNA breaks and interstrand crosslink damage repair through the use of sister chromatids as a repair template. BRCA1/2 are critically important proteins in this pathway. HR deficiency (D) is the result of germline or somatic genetic alterations in HR genes (i.e., BRCA 1 or 2) [5]. Dysfunctional HR genes cause genome-wide errors and can lead to tumorigenesis [6, 7]. Tumors that are not HRD are considered HR proficient (P) and contain no functional genetic alterations in HR pathway genes, like BRCA1/2, resulting in faithful DNA repair, thereby reducing the mutation burden. While the HR pathway is responsible for repairing double-stranded breaks, the base excision repair pathway repairs single-stranded DNA breaks. Poly (ADP-ribose) polymerase proteins (PARPs) are essential proteins in this pathway. When PARPs are inhibited, single-stranded breaks are converted to double-stranded breaks during DNA replication. Synthetic lethality occurs in cells treated with a PARP inhibitor that have a *BRCA* mutation or are HRD. 

Alterations in HR pathway genes, especially mutations in BRCA1/2, can be germline and confer familial risk for breast, ovarian, prostate, and pancreatic cancer [8] or somatic. For patients who demonstrate negative germline testing, somatic HR molecular status is assessed by NGS and is most commonly evaluated by Myriad’s MyChoice CDx-testing. This involves the analysis of BRCA 1 and 2 gene mutation status, loss of heterozygosity (LOH), telomeric allelic imbalance (TAI), and large-scale state transition score (LST) to determine a genomic instability score (GIS) [9]. Each is weighted and scored using a proprietary algorithm to determine the level of genomic instability. A GIS ≥42 in BRCA 1 or 2 negative patients defines HRD status. A GIS score <42 defines HRP status [10]. BRCA 1 or 2 mutations or HRD molecular profile tumors are a sensitive ovarian cancer population to PARP inhibitor therapy [10–14] and are associated with a better prognosis in patients receiving platinum-based chemotherapy and/or bevacizumab [15]. However, ovarian cancer patients with HRP molecular status have a worse prognosis with standard-of-care therapy involving PARPIs, bevacizumab, and platinum-based chemotherapy [11, 16–19]. This is related to the ability of HRP molecular status tumors to perform DNA repair, resulting in decreased DNA damage induced by these therapeutic options. Regardless of mutation status, all tumors demonstrate benefit from frontline maintenance treatment with PARP inhibitors, although the magnitude of benefit is greater in patients with a BRCA mutation or HRD, as demonstrated in multiple clinical trials [11, 12]. However, multiple resistance mechanisms have been demonstrated. Additionally, PARP inhibitors in the recurrent setting have recently been shown to be detrimental to OS, and the FDA has removed them from this setting in patients without a BRCA mutation^1^ [20, 21]. Additional studies are underway investigating PARP inhibitor combination therapy to overcome the limitations of PARP inhibitor therapy [22]. Results are expected soon from several large Phase 3 clinical trials evaluating combination PARP inhibitor and checkpoint inhibitor therapy. Previous studies evaluating checkpoint inhibitors in ovarian cancer have been largely negative [23, 24].

## Vigil

Vigil is a novel, triple-function, cell-based immunotherapy recently cleared by the FDA for the initiation of a phase 3 registration trial in newly diagnosed stage IIIb/IV HRP-positive ovarian cancer patients. Vigil expresses GMCSF, an immunostimulatory cytokine, and a bifunctional short-hairpin RNA to knockdown furin. Furin is the critical convertase responsible for the cleavage of TGFβ1 and 2. Finally, Vigil provides the full complement of personal neoantigens relevant to the patient’s cancer, allowing for T cell education and priming. Data supporting the efficacy of Vigil in the ovarian cancer population include results from phase 1, 2a, and 2b trials [25–31].

A phase 2b, double-blind placebo-controlled trial [25] recently evaluated 91 newly diagnosed stage IIIb-IV ovarian cancer patients randomized to Vigil vs. placebo at maintenance following debulking surgery and combination cisplatin/taxane induction chemotherapy. The molecular profiles of the 91 patients enrolled in the study included BRCA-mutant, HRD, and HRP patients. At the time of trial initiation and accrual, the use of somatic HRD/HRP testing was not part of clinical practice, so somatic testing was not done prospectively. A *post hoc* analysis demonstrated improved clinical benefit correlated with increased DNA repair capacity (HRP) with Vigil. A trend toward clinical benefit in RFS was observed in all patients (HR 0.688, *p* = 0.078) [25]. However, a statistically significant survival benefit was observed in the non-germline mutated population (combination of HRD and HRP) in both RFS and OS (HR 0.514, *p* = 0.020; HR 0.493, *p* = 0.049, respectively). The HRD/HRP subgroups were evaluated using Myriad’s MyChoice CDx. The greatest benefit following Vigil treatment was observed in those patients with the highest capacity for DNA repair and those with the HRP molecular profile, in both RFS and OS (HR 0.386, *p* = 0.007 and HR 0.342, *p* = 0.019, respectively) [26, 27]. This effect was durable and continued at a long-term follow-up of 3 years [27].

## Discussion

**FIGURE 1:**
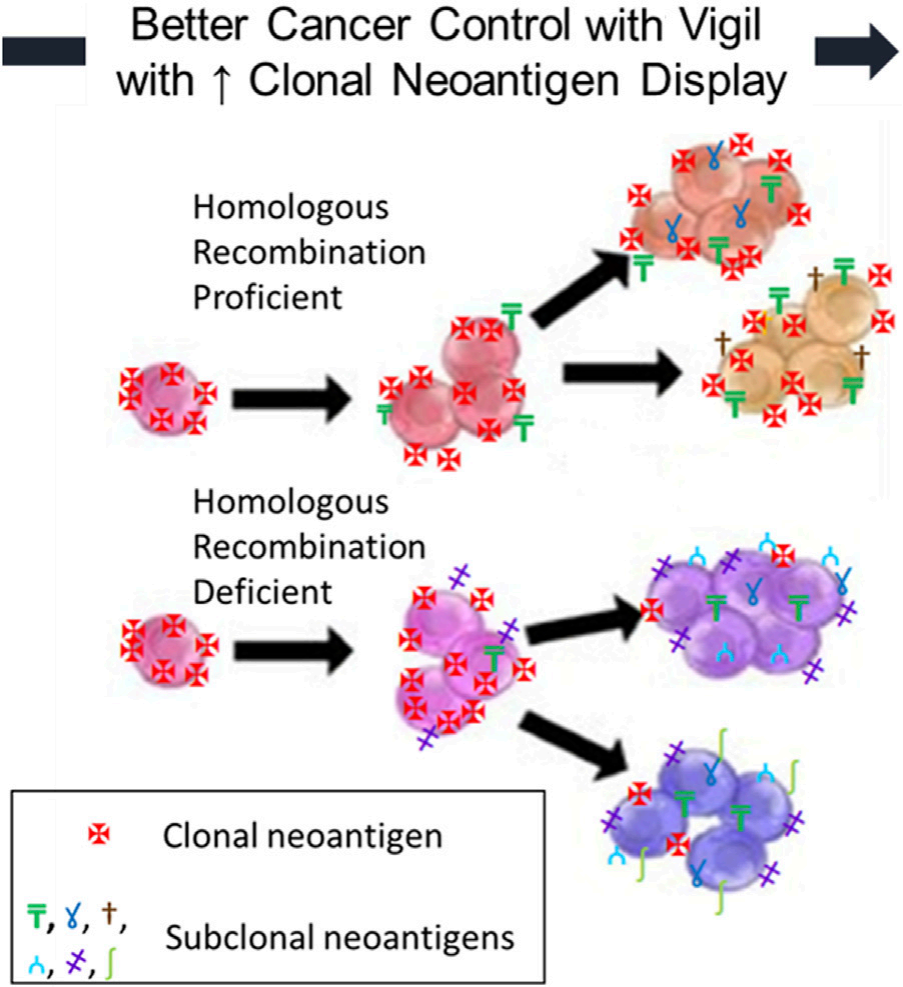
Homologous Recombination Deficient (HRD) and Proficient (HRP) OvCs: neoantigen fraction (clonal vs. subclonal) affects immunogenicity. The clinical benefit of Vigil is improved with increased clonal neoantigen display.

Recent literature, both preclinical and clinical, has convincingly demonstrated the role of clonal neoantigen burden in correlating OS improvement with checkpoint inhibitor therapy in advanced cancer patients [32–34]. CD8^+^ lymphocytes reactive to clonal neoantigens have been identified in multiple studies [32–36]. Durable clinical benefit has been correlated with the identification of T cells recognizing clonal neoantigens (not subclonal neoantigens) [32–36]. Cytotoxic chemotherapy has been shown to increase the proportion of subclonal neoantigens and reduce the expression and visibility of clonal neoantigens, thereby reducing the responsiveness of malignant cells to immunotherapy [32, 34, 35]. The inconsistency of PD-L1 expression and checkpoint inhibitor response appears to be related to the proportion of clonal neoantigens targeting effector cells. We hypothesized that Vigil activity would be more likely to provide clinical benefit in patient tumors with higher expression of clonal neoantigens (present on all tumor cells) as opposed to subclonal neoantigens (only present on newly mutated subpopulations). It is our premise that Vigil construction involving autologous tumors and *ex vivo* transfection with a dual plasmid containing bi-shRNAi furin/GMCSF wild-type DNA would generate a more active clonal neoantigen profile to induce an effector cell response in malignant cells containing the HRP molecular profile [37–40]. In this scenario, the clonally matched targets are more highly visible and associated with greater effector cell responsiveness as compared to effector cells impacting subclonal neoantigens that are selectively on tumor cells (Figure 1). This effect has been demonstrated in the BRCA-wt HRP population clinical benefit to Vigil.

In conclusion, the achievement of high clonal neoantigen targeting capacity provides an enhancement in immunotherapeutic proficiency and is likely induced by Vigil treatment and optimized by HRP molecular profile capacity.

## Footnotes

1 Clovis Oncology. Rubraca Rucaparib for Treatment of BRCA-Mutated Ovarian Cancer after 2 or More Chemotherapies Is Voluntarily Withdrawn in the U.S. Health Care Provider Letter (2022).
